# Elective Sternotomy With Fluoroscopic Guidance for the Removal of a Residual Implantable Cardioverter Defibrillator Lead: A Case Report and Literature Review

**DOI:** 10.7759/cureus.77148

**Published:** 2025-01-08

**Authors:** Said Khallikane, Nabil Mehdi, Mehdi Didi, Hicham Kbiri, Youssef Qamouss

**Affiliations:** 1 Anesthesiology and Critical Care, Military Hospital of Avicenne, Marrakech, MAR; 2 Anesthesiology and Critical Care, Faculty of Medicine and Pharmacy of Rabat, Rabat, MAR; 3 Anesthesiology and Critical Care, Mohammed V Military Instruction Hospital, Rabat, MAR; 4 Anesthesiology, Intensive Care Unit, and Emergency, Military Hospital of Avicenne, Marrakech, MAR; 5 Anesthesiology and Reanimation, Military Hospital of Avicenne, Marrakech, MAR

**Keywords:** cardiac, elective sternotomy, esophageal achalasia, fluoroscopic guidance, infective endocarditis, intramyocardial lead removal, leads in innominate vein, residual defibrillator leads, rhythm-related complications, transesophageal echocardiography contraindications

## Abstract

Infectious myocarditis (IM) and infective endocarditis (IE), sometimes associated with infection of the surrounding mediastinal tissue or embolic complications caused by residual implantable cardioverter defibrillator (ICD) lead material embedded in the ventricle, present a significant challenge for cardiac surgeons due to the difficulty of precisely locating the old intracardiac pacing lead remnants because of the heart's continuous movement. We present the case of successful two-stage elective sternotomy extraction of two residual defibrillator leads, one trapped in the left innominate vein, easily removed after veinotomy without cardiopulmonary bypass (CPB), and the other embedded intramyocardially in the inferior wall of the right ventricle, successfully removed under CPB after fluoroscopic guidance. The patient was discharged four weeks post-operation without complications. In our case, transesophageal echocardiography (TEE) was not performed due to the patient's history of esophageal achalasia.

## Introduction

Residual implantable cardioverter defibrillator (ICD) lead material embedded in the right ventricle is rarely seen in clinical practice. It is a highly life-threatening situation, with a high incidence of dying from infectious myocarditis (IM), infective endocarditis (IE), septic shock, valve destruction, pulmonary embolism, and systemic embolism or tamponade due to perforation caused by heart movements [1,2]. The ends of stimulation lead are encased in the myocardium, either during a spontaneous rupture of the leads or during transvenous extraction maneuvers to treat IE on implantable devices such as cardioverter defibrillators or pacemakers, representing a major challenge for surgeons, as it is difficult to precisely locate the lead tips embedded in the myocardium due to the continuous movement of the heart, especially if they induce no extramyocardial or intramyocardial reaction [2-4]. Here, we present an illustrative case of removal of an old ventricular pacing lead tip embedded in the myocardium in a young man who developed IE with sepsis, an anterior mediastinitis, and subsequent dehiscence of the anterior sternotomy wound with pus drainage along fistulous tracts. The procedure was performed via sternotomy under cardiopulmonary bypass (CPB), guided by fluoroscopy, instead of transesophageal echocardiography (TEE). Fluoroscopic guidance showed significant advantages over TEE in accurately locating the lead tip in the myocardium, avoiding the traumatic passage of a TEE probe in a patient with stage II to III achalasia. After multiple unsuccessful esophageal dilations, a multidisciplinary team decided against TEE, which is contraindicated due to the high risk of perforation in advanced achalasia. This patient's condition necessitated steering away from any esophageal interventions, leading to the use of fluoroscopy. Fluoroscopy effectively facilitated lead localization while preventing complications from blind probing, though it cannot detect issues like myocardial perforation or embolism as effectively as echographic modalities. Additionally, it involves radiation exposure and offers limited monitoring. In our case, thoracoscopy wasn't suitable due to the intervention's nature, requiring direct visualization of the deeply embedded lead in the myocardium. The use of TEE, or transthoracic echocardiogram (TTE), is typically employed to avoid thoracotomy and the need for CPB during minimally invasive surgeries, such as thoracoscopy in similar cases.

## Case presentation

**Figure 1:**
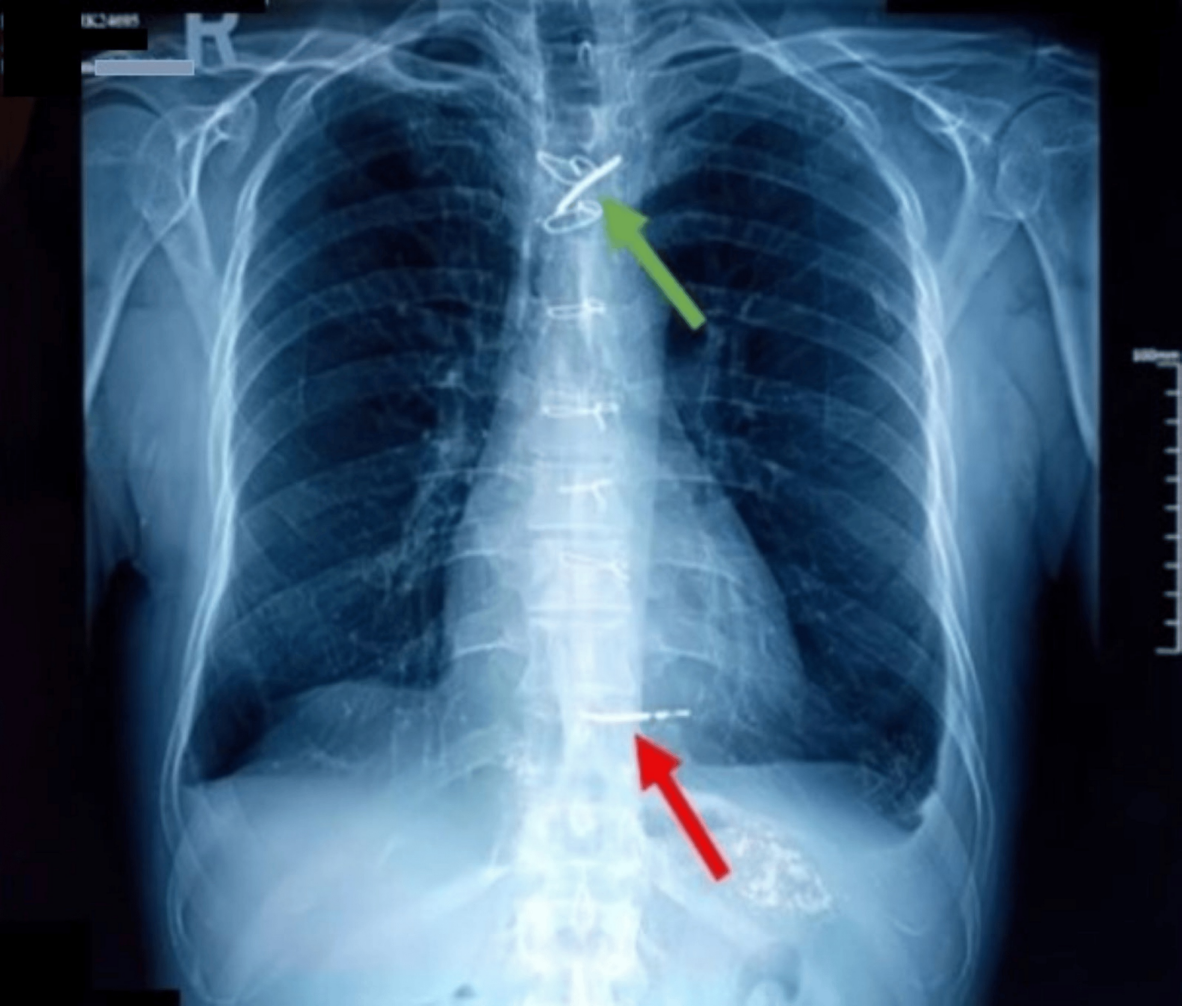
Chest X-ray (frontal view) showing two radio-opaque objects corresponding to the tips of the ventricular lead. The upper tip is projected over the left innominate vein (green arrow), and the lower tip is projected over the right ventricle (red arrow)

**Figure 2:**
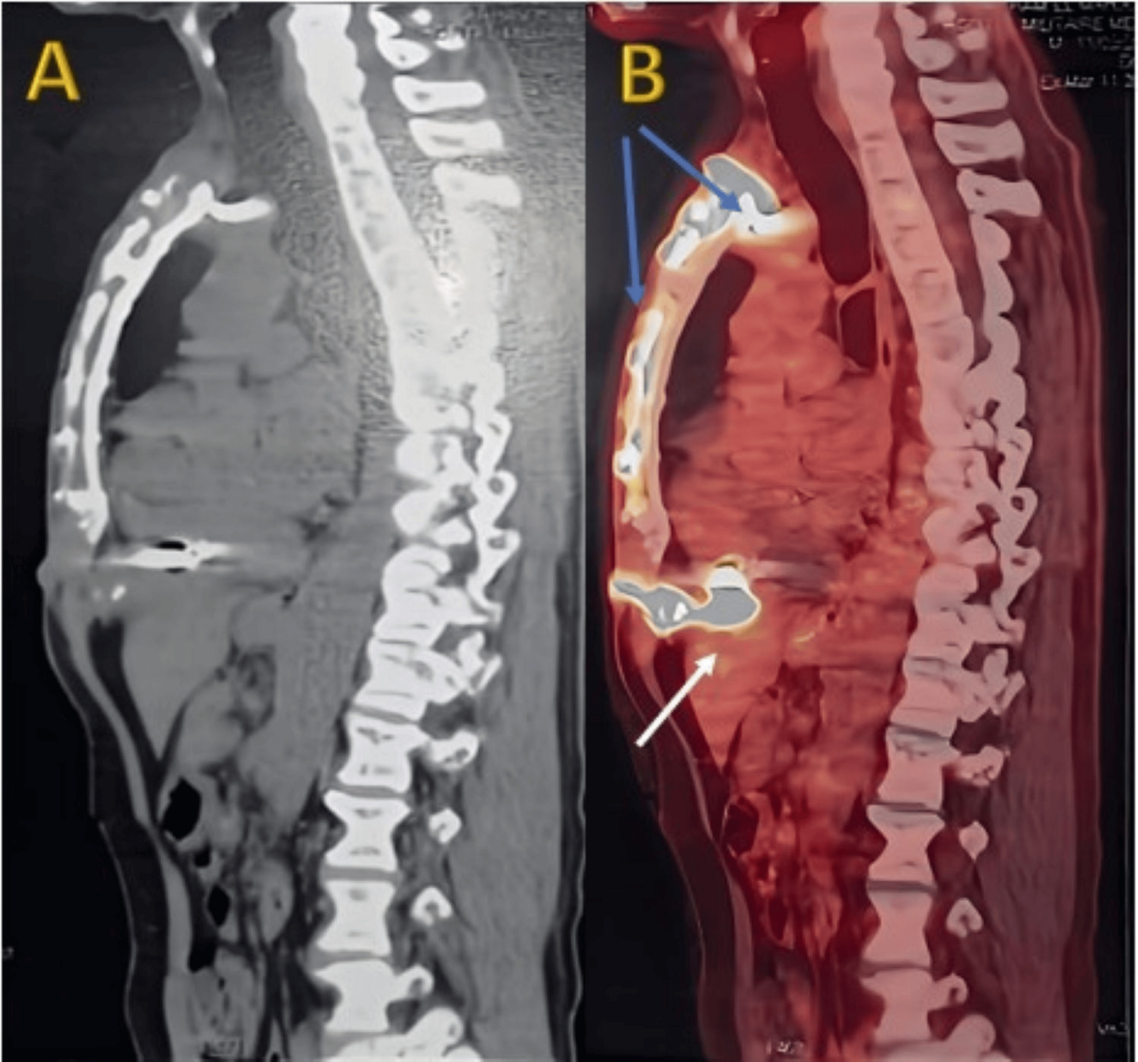
(18F)-FDG PET scan revealing pathological hypermetabolism and associated findings in cervicothoracoabdominal sections On PET scan with (18F)-FDG imaging in sagittal cervicothoracoabdominal sections, prior to tracer injection (A), retrosternal material is observed extending into the left subclavian region (proximal end of the ICD lead), projecting over the subclavian vein and left innominate vein. Additionally, hyperdense material (right ventricular lead tip) generates artifacts at the apex of the right ventricle. One hour after (18F)-FDG tracer injection (B), intense pathological hypermetabolism is noted in the left supraclavicular region along a muscular trajectory and retrosternally, extending into the left subclavian area near the innominate vein, likely of infectious origin. This is associated with linear pathological hypermetabolism involving the manubrium and sternal body (blue arrows), extending superiorly to a pathological median suprasternal cutaneous hypermetabolism and inferiorly to hypermetabolism below the sternal notch. It further extends intrathoracically at the level of the right ventricle, adjacent to the residual material (white arrow). FDG: fluorodeoxyglucose; ICD: implantable cardioverter defibrillator

**Figure 3:**
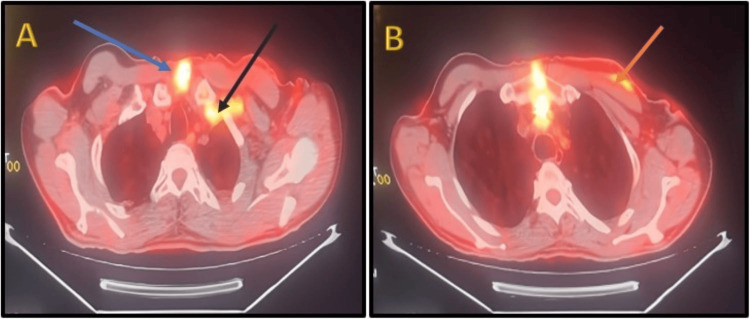
(18F)-FDG PET scan showing pathological hypermetabolism in cervicothoracic sections On PET scan (18F)-FDG imaging in a transverse cervicothoracic section, showing pathological cutaneous hypermetabolism in the median suprasternal region (blue arrow), extending into the left subclavian area over the innominate vein, which also shows pathological hypermetabolism (black arrow) (A). This hypermetabolism extends anteriorly along the K1 arc and beneath the pectoralis muscle (orange arrow) (B). FDG: fluorodeoxyglucose

A 64-year-old male active smoker, weighing 65 kg, was equipped with a single-chamber ICD in 2006 due to recurrent monomorphic sustained ventricular tachycardia despite antiarrhythmic therapy escalation. The arrhythmia occurred following myocardial infarction without obstructive coronary arteries (MINOCA) caused by coronary artery spasms. In 2019, the ICD was upgraded to a dual-chamber device, with the addition of an atrial lead and generator replacement at the end of its life. The patient presented with chills, fever, and signs of infection at the generator pocket site, without heart murmurs, and signs of heart failure with persistent fever. Laboratory results showed significant inflammatory markers (CRP of 110 mg/L). Initial blood cultures were negative, but repeated cultures revealed *Staphylococcus aureus* resistant to methicillin. TTE detected a 1 cm vegetation attached to the atrial lead tip, with no tricuspid valve involvement. TEE could not be performed as the patient is being treated for achalasia of the esophagus. Chest CT excluded septic pulmonary embolism. The diagnosis was lead-associated IE with severe sepsis. Antibiotic therapy with vancomycin, gentamicin, and rifampin was initiated. Percutaneous removal of the generator and atrial lead was performed on day 5, but the ventricular lead could only be partially removed due to its long-standing placement, despite the use of a laser sheath. Persistent sepsis led to performing sternotomy under CPB during the occurrence of another episode of IE associated with septic pulmonary embolism in August 2022 for the extraction of the ventricular lead and the vegetations from the infected portion of the atrial wall and the implantation base of the anterior leaflet of the tricuspid valve. However, it also failed to fully remove all material due to the lead's adherence to the right ventricular wall. The lead was released from the free edge of the tricuspid valve and embedded through the posterior pillar at the inferior wall level. It was then cut flush with its embedding in the myocardium. The immediate postoperative course was favorable, with clearance of sepsis, negative blood cultures, and normalization of inflammatory markers. The patient was discharged after completing six weeks of antibiotics. However, three months later, progressive dehiscence of the sternotomy wound with purulent drainage was noted. Imaging, chest X-ray (Figure 1), contrast-enhanced CT, and PET-CT (Figures 2-3) showed residual material in the left brachiocephalic vein and near the right ventricular wall, with associated soft tissue infection in the sternum and left pectoral region but no evidence of persistent IE on TTE or clinical grounds.

**Figure 4:**
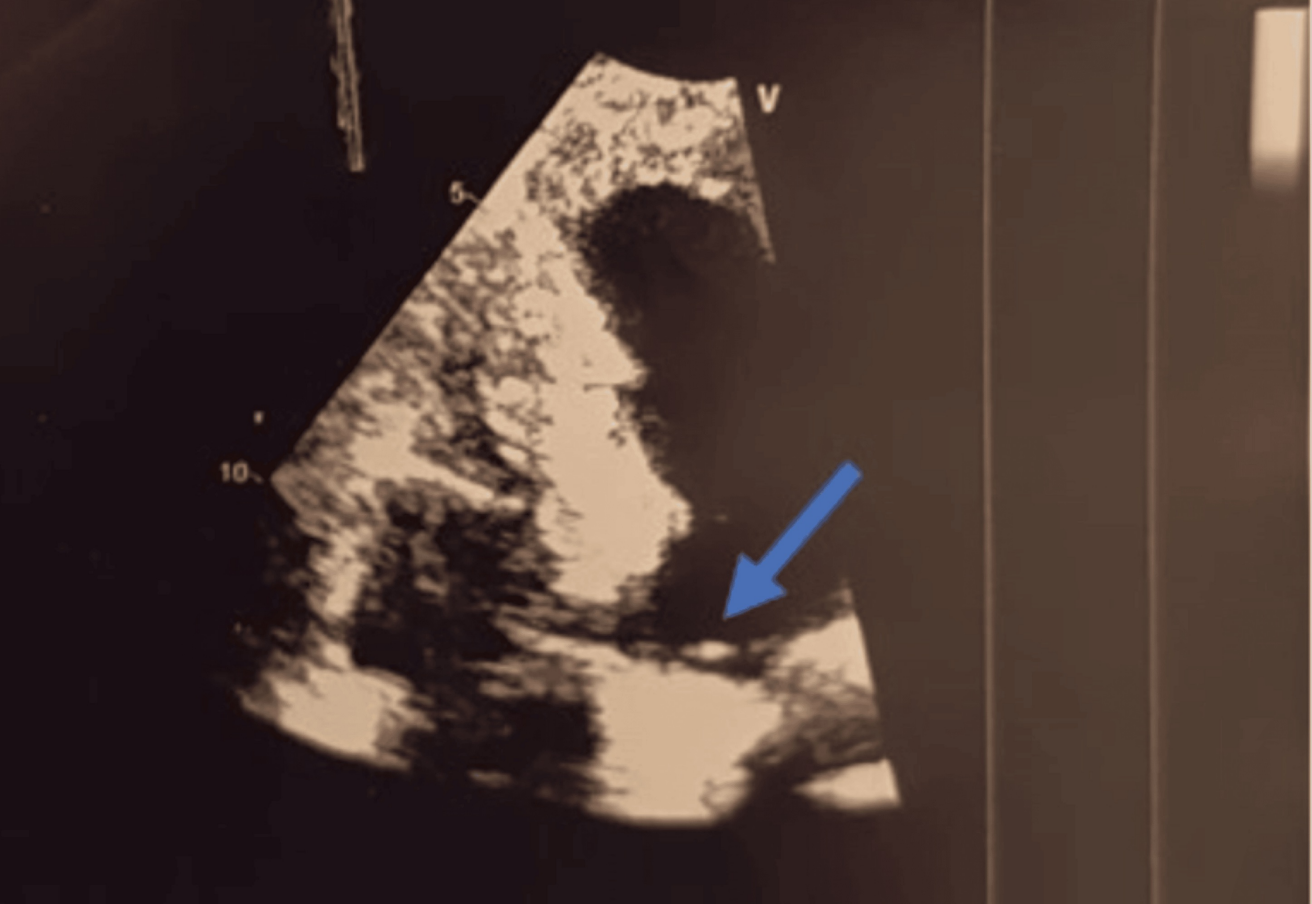
Echocardiogram image showing a non-dilated, non-hypertrophied left ventricle (LV), with an element at the base of the right ventricle (RV) compatible with a tip of an ICD lead (blue arrow) ICD: implantable cardioverter defibrillator

The patient self-medicated with intermittent ciprofloxacin, which temporarily reduced wound drainage during febrile episodes. In November 2024, he presented with fever, chills, and a draining fistula at the xiphoid portion of the sternotomy scar. Preoperative TTE showed no vegetation or valvular involvement, with preserved cardiac function, except for a foreign body embedded at the base of the right ventricle (Figure 4); however, TEE was contraindicated. Repeated blood cultures were negative due to the patient's prior continuous use of antibiotics.

**Figure 5:**
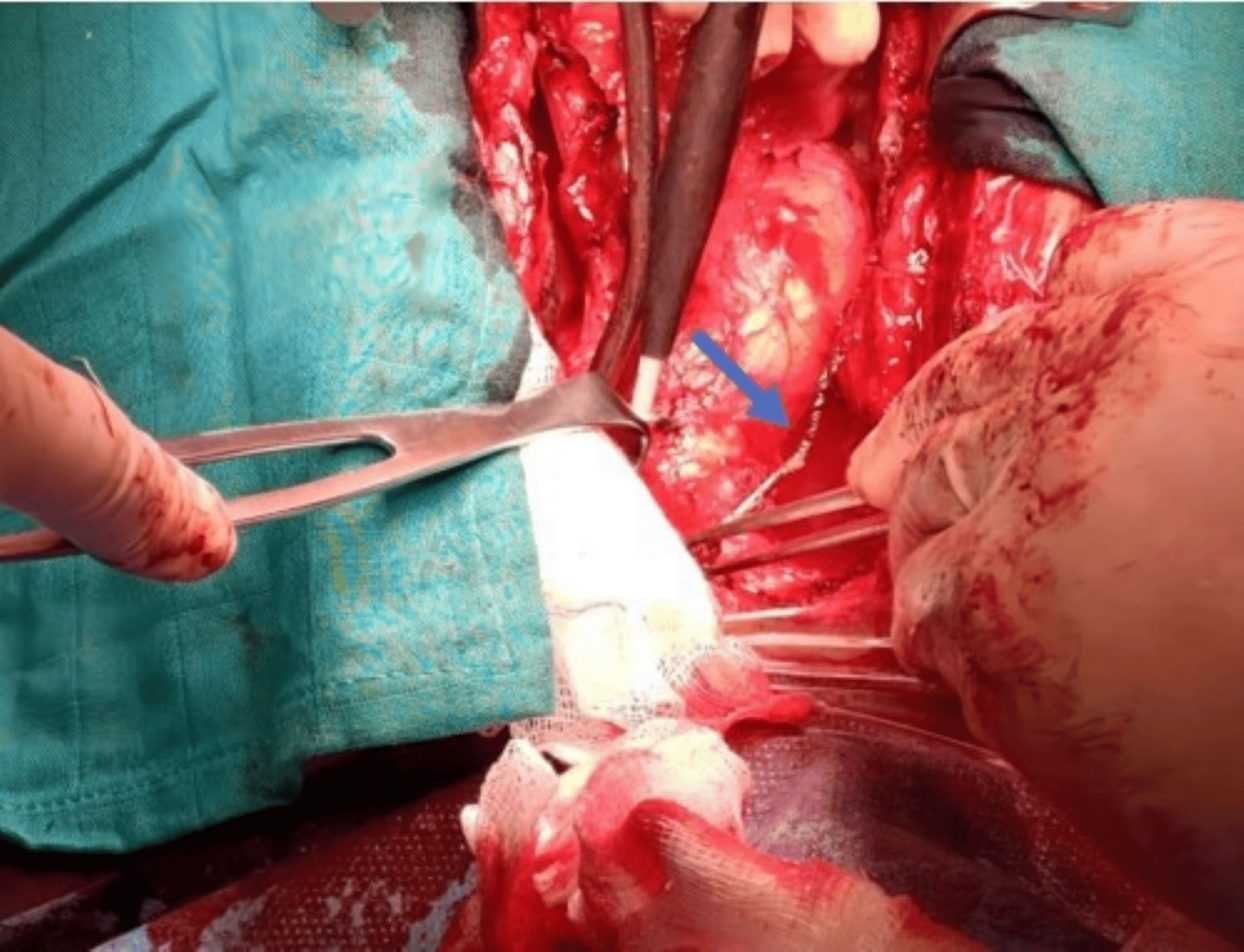
Intraoperative photo showing the extraction using forceps, aided by adequate exposure of the left subclavian region with a retractor, from the frayed and tangled wire (blue arrow) of the ventricular lead tip that was partially retained with its sheath inside the left innominate vein through a venotomy performed with an electric scalpel

**Figure 6:**
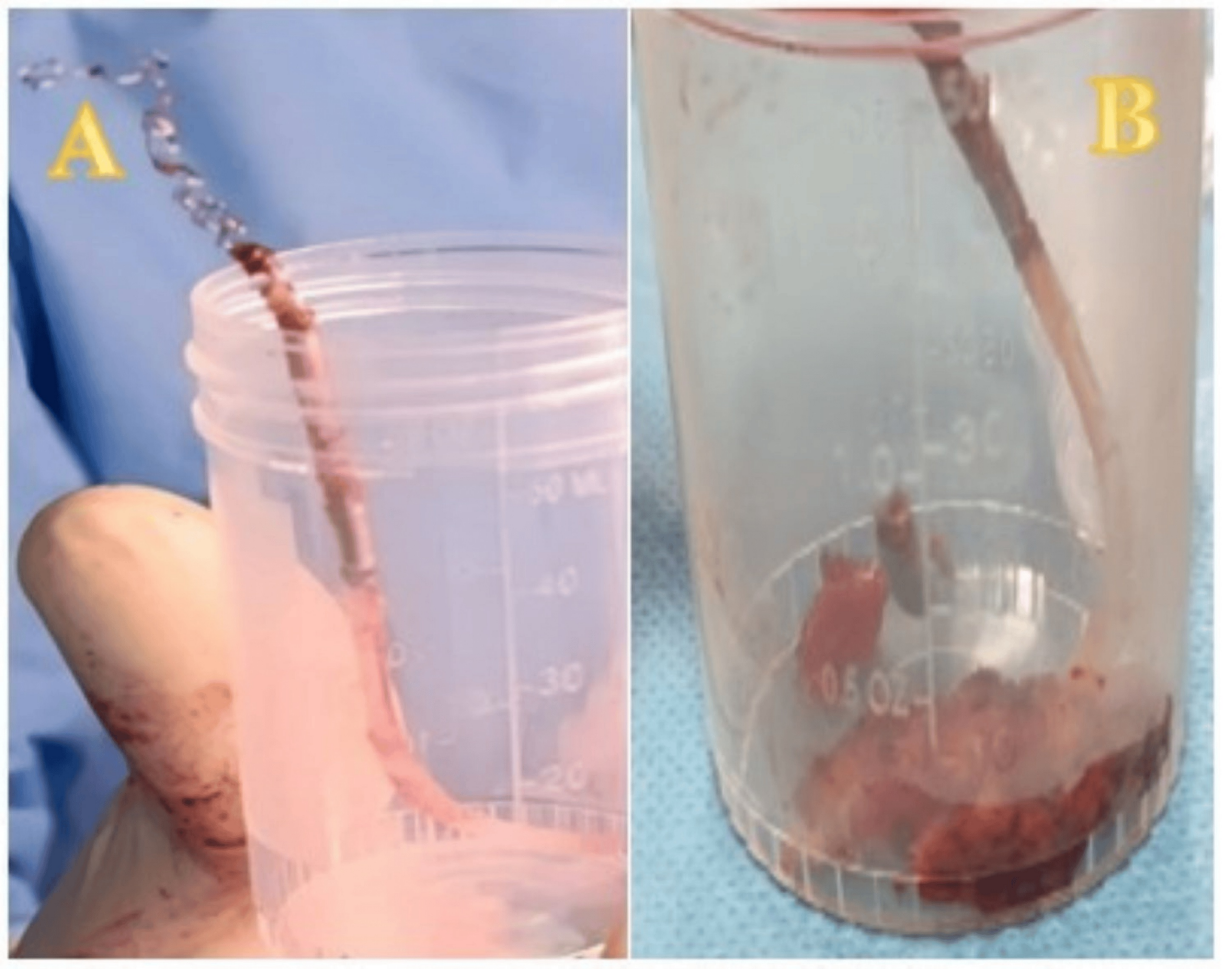
Photo showing the ventricular lead tip with its sheath and tangled wires, deformed after successful extraction from the left innominate vein (A), along with surrounding infected mediastinal tissue and fragments of thrombosis (B). The specimen was placed in a sterile container for cytobacteriological and histopathological studies

However, due to the fixation of two foreign bodies on PET scan imaging and the presence of sepsis with purulent drainage from the sternotomy wound, further intervention was warranted. Multidisciplinary evaluation deemed the surgical risk acceptable. A combined surgical approach was planned, involving sternotomy without CPB to address the left brachiocephalic vein, followed by right atriotomy under CPB to extract the residual ventricular lead. Intraoperative findings included two fistulous tracts filled with pus: the first one extending from the pocket site to a thrombosed left innominate vein containing an encased ventricular lead filled with pus, coiled and attached to the vein wall (Figure 5), which was removed after venotomy without the need for veinoplasty and was placed in a sterile container for cytobacteriological analysis (Figure 6), and the second one extending from the sternal notch to the base of the right ventricle.

**Figure 7:**
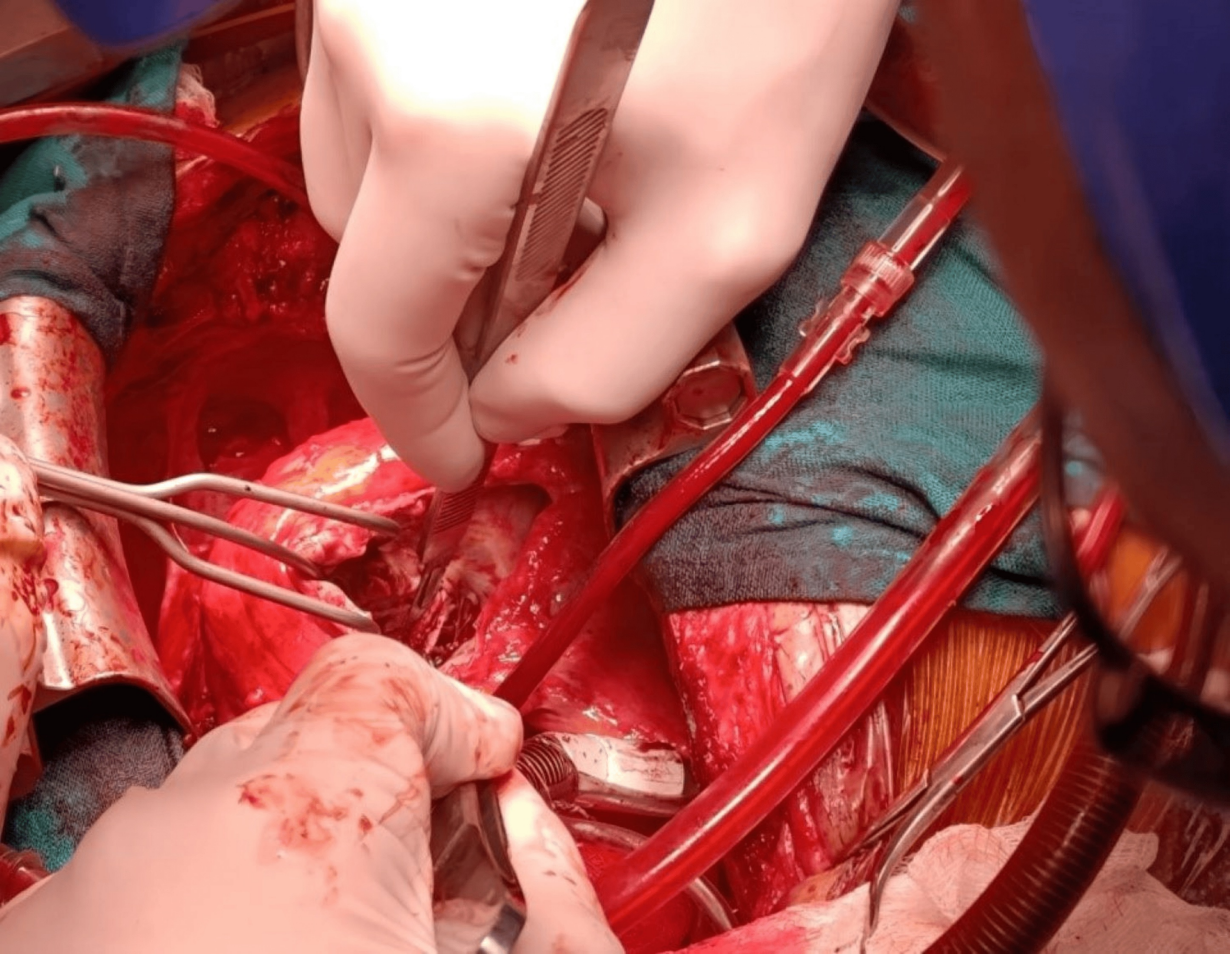
Intraoperative image during the second stage of surgery under cardiopulmonary bypass (CPB) with clamping, showing a right atriotomy to begin the extraction of the ventricular lead embedded in the muscular wall through the posterior pillar at the inferior wall level, following radiographic localization

The remaining ventricular lead was embedded in the right ventricular wall, firmly adherent, after right atriotomy under CPB (Figure 7) from a site showing localized infectious signs. After several blind unsuccessful extraction attempts based on previous imaging data, and to avoid causing further damage, fluoroscopy was utilized with iterative adjustments of angles and localization using forceps.

**Figure 8:**
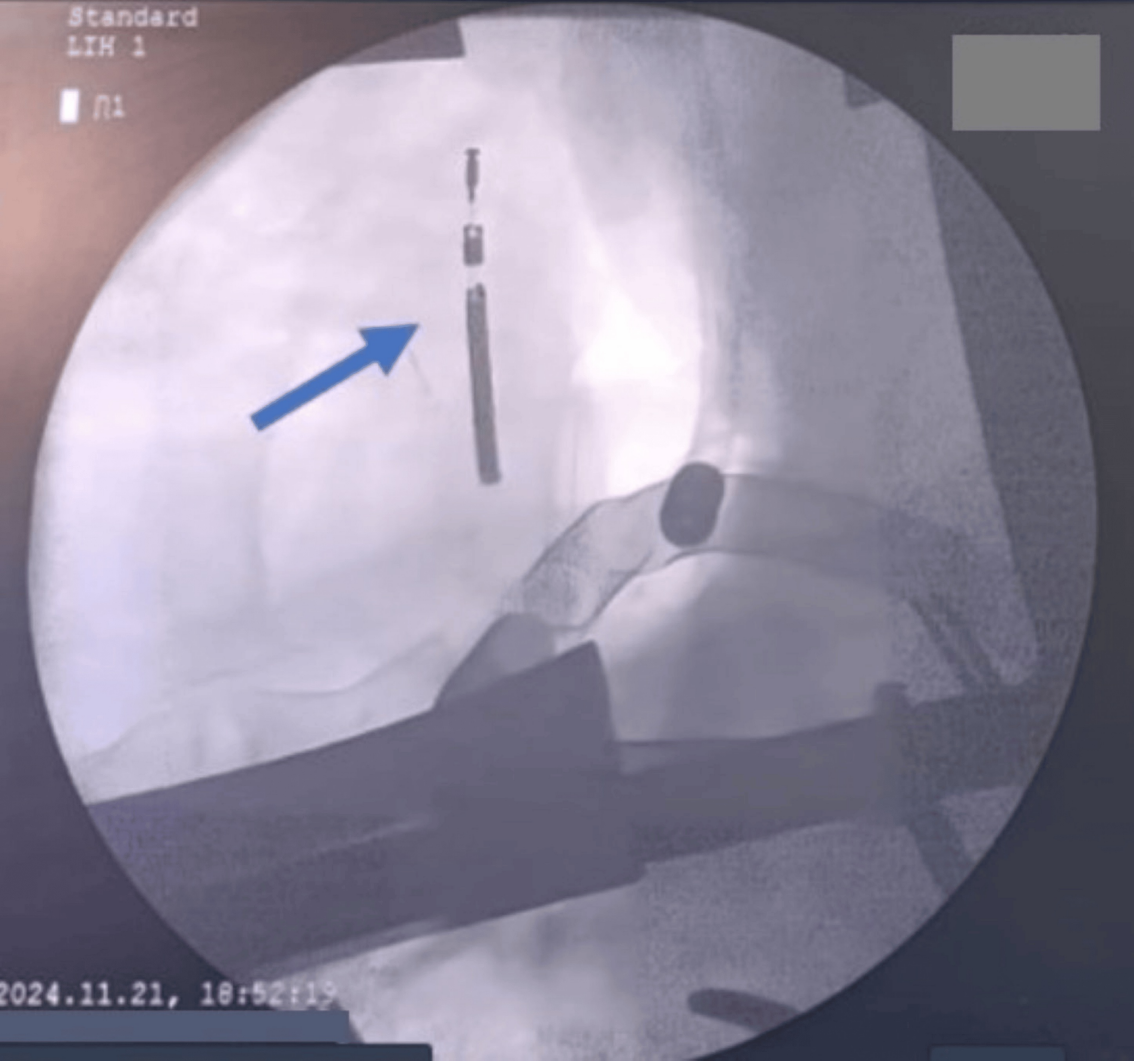
Intraoperative fluoroscopic image of the right ventricle showing the ventricular lead tip (blue arrow), cut and embedded in its inferior wall, serving as a guide for surgical extraction

**Figure 9:**
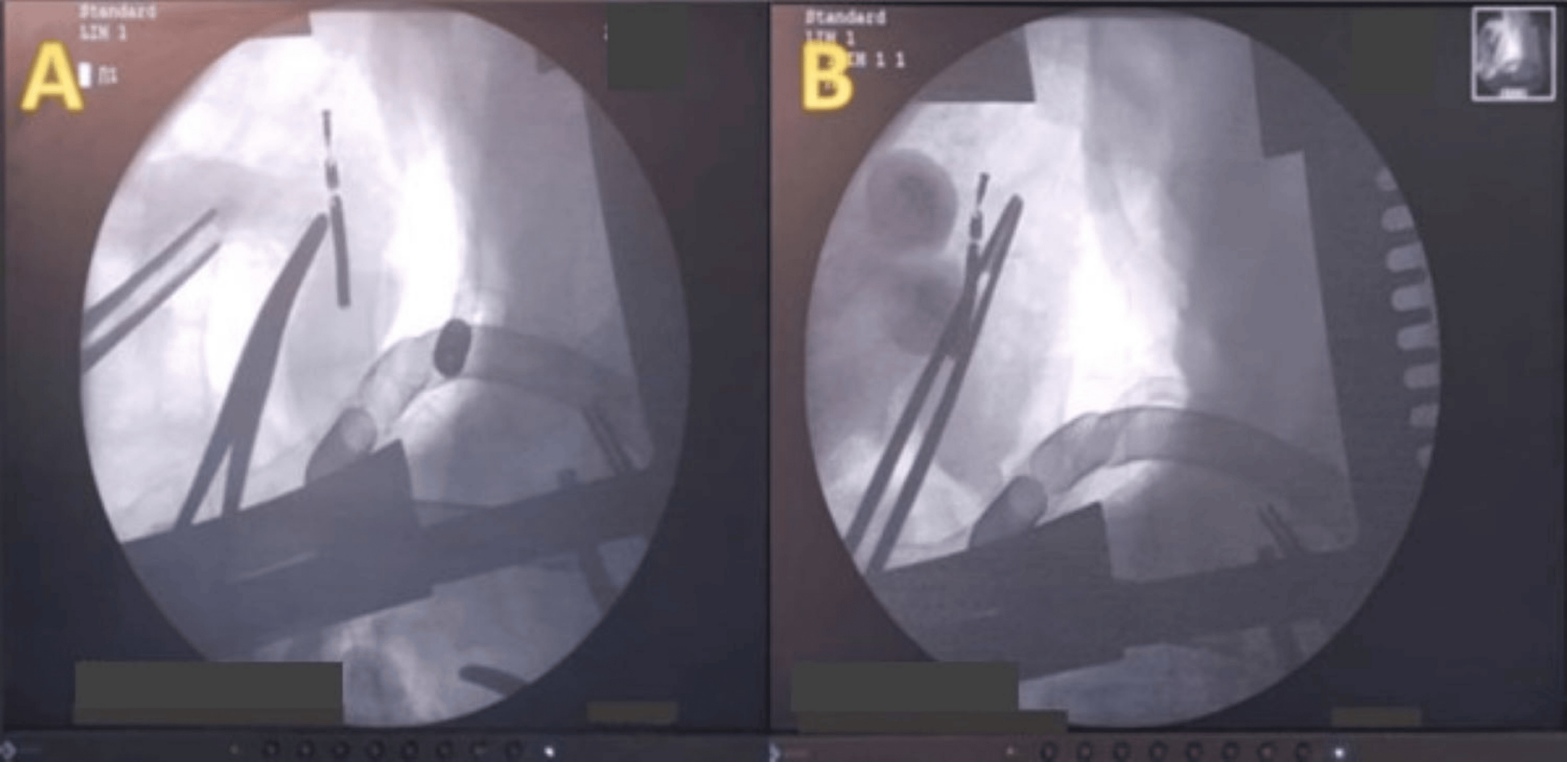
Intraoperative fluoroscopic image centered on the right ventricle, showing the cut ventricular lead tip inside the right myocardium, with the exposure of instruments serving as landmarks for surgical extraction (A), as well as the progression of the forceps through the endocardium before grasping the lead (B)

**Figure 10:**
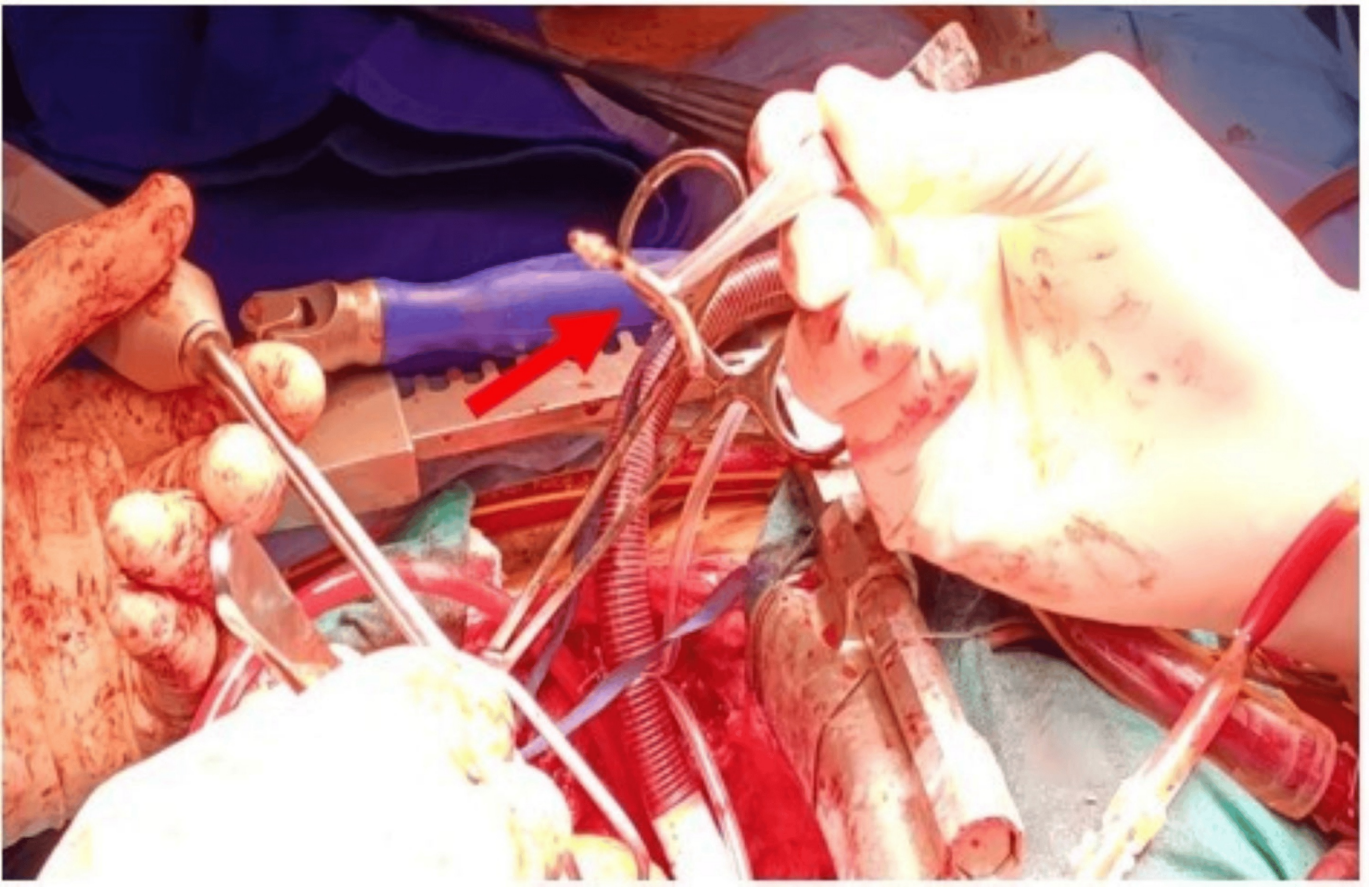
Intraoperative photo showing the tip of the right ventricular lead (red arrow) after its extraction, following fluoroscopic localization

Finally, the lead was extracted under fluoroscopic guidance (Figures 8-9) in the form of metal corresponding to the tip of the ventricular lead (Figure 10), instead of TEE, which its use was contraindicated in our patient due to his history of esophageal achalasia.

**Figure 11:**
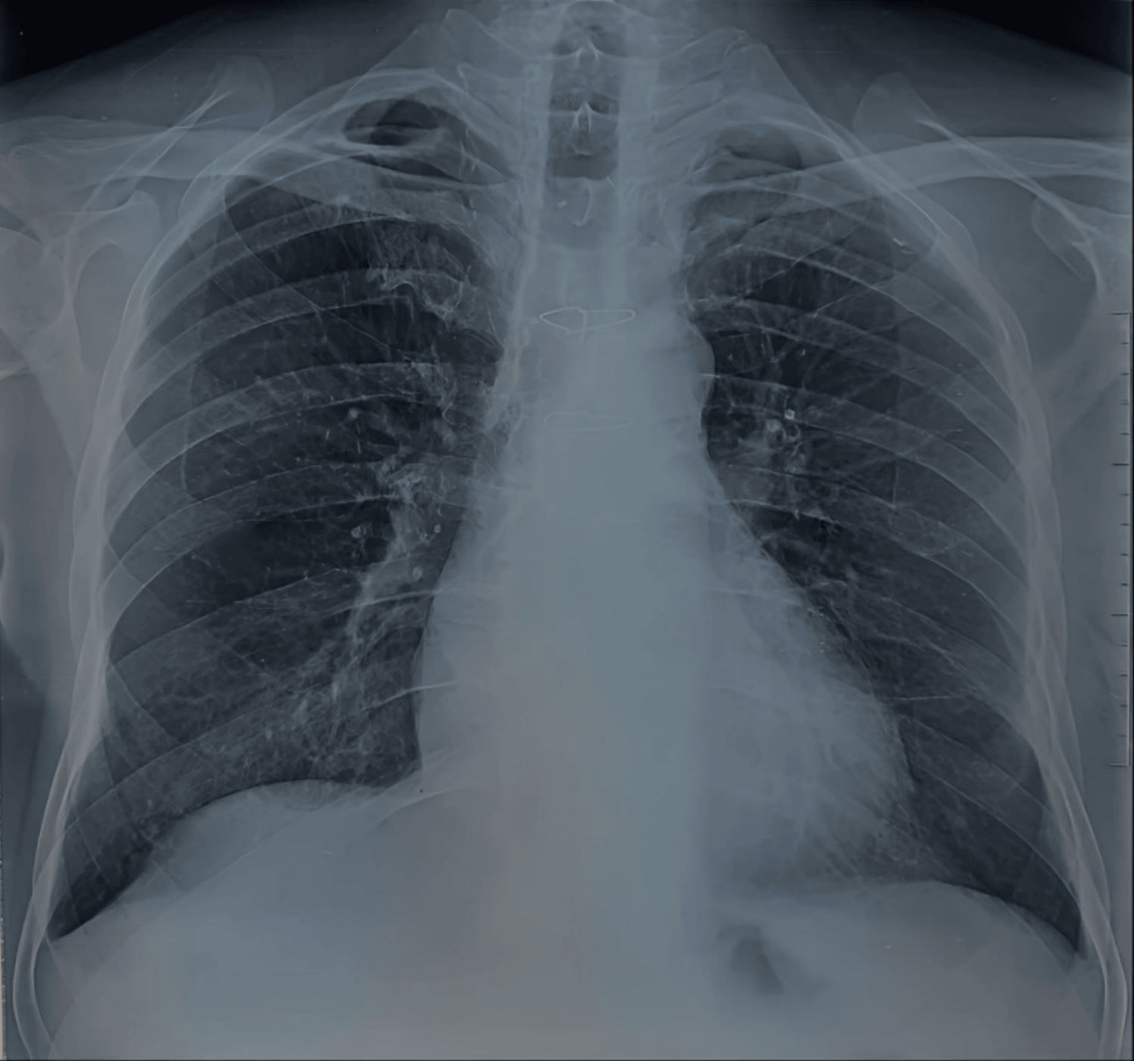
Postoperative day 7 chest X-ray showing the disappearance of the material at both the level of the innominate vein and the right ventricle, as well as the regression of the left pleural effusion

The diagnosis of IM was made. However, bacteriological analysis of both ends of the lead and the surrounding tissues revealed no microorganisms upon direct examination and culture. Nevertheless, there were no signs of intracardiac or valvular infection. The lack of microorganisms in the cultures can be attributed to the patient's prior self-medication with antibiotics. However, the diagnosis of IM was established through a comprehensive evaluation that incorporated clinical assessments, intraoperative findings, imaging results, and histopathological analysis. This analysis substantiated the presence of a significant inflammatory response, as evidenced in both the pus and the thrombotic and necrotic tissues that were extracted. Postoperative recovery was uneventful, and the patient completed a four weeks course of antibiotics with a postoperative day 7 chest X-ray (Figure 11) showing the disappearance of the material at both the level of the innominate vein and the right ventricle, as well as the regression of the left pleural effusion, without complications, the infectious and inflammatory biomarkers returned to normal throughout the hospitalization, notably with the normalization of CRP levels.

This case highlights the effectiveness of fluoroscopy-guided surgery for extracting myocardial-embedded lead fragments when standard echocardiographic methods like TEE or TTE are inaccessible or contraindicated. It enables real-time visualization and localization of radiopaque objects, ensuring the safe removal of intramyocardial foreign bodies. Due to the contraindication of TEE, the fluoroscopy thus represents a valuable alternative in complex cases, minimizing complications and also facilitating the effective extraction of self-inflicted intramyocardial foreign materials; it proves useful, especially in isolated cases of foreign body extraction from the myocardium. This underscores the importance of multidisciplinary management and tailored therapeutic strategies. Surgical extraction of infected leads is a formal recommendation, but the timing and approach remain challenging, especially for patients dependent on ICD therapy. This underscores the importance of multidisciplinary management and tailored therapeutic strategies and emphasizes the complexity of chronic lead infections, particularly in patients requiring multiple generator or lead replacements, increasing the risk of infection and complicating therapeutic decisions.

## Discussion

The clinical presentation of cardiac implantable electronic device (CIED)-associated IE often resembles valvular IE, with symptoms like fever, chills, and embolic events. Signs of pocket infection, such as swelling or discharge, may be absent.

Since 2000, the modified Duke criteria have shown 80% sensitivity in diagnosing IE, but they struggle with prosthetic materials, grafts, and cardiac devices, as echocardiography is inconclusive in 30% of cases [5].

The 2015 ESC criteria improved diagnostic accuracy through a multimodal imaging approach, incorporating echocardiography, CT, MRI, and PET/CT [6].

TTE and TOE are recommended for suspected CIED-related IE, with ICE as an alternative if TOE isn't possible. The absence of vegetation doesn't rule out IE, as they may be on unseen extracardiac segments. Repeat TTE or TOE after 5-7 days if initial results are negative but suspicion persists. Fibrinous lead masses can appear in asymptomatic CIED patients and don't predict long-term IE [7].

Blood cultures are essential for diagnosing IE, but a single positive culture should be interpreted with caution. Blood culture-negative endocarditis (BCNIE) often results from prior antibiotic treatment or rare pathogens, requiring repeated blood cultures, serological testing, and PCR analyses [8]. Samples must be taken before initiating antibiotic treatment. *S. aureus* bacteremia suggests IE related to a CIED, while Gram-negative bacteremia is associated with visible pocket infection. Biomarkers such as CRP and procalcitonin, although useful for assessing sepsis and monitoring treatment, lack specificity for diagnosing IE [9,10].

The management of infections associated with CIEDs necessitates the complete removal of the device, as conservative management has been associated with an increase in mortality rates, along with the initiation of empirical antibiotic therapy based on guidelines that target methicillin-resistant *S. aureus* (MRSA) and Gram-negative bacteria, which is subsequently followed by pathogen-specific therapy. In instances where total device removal is not feasible, a course of intravenous antibiotic treatment lasting four to six weeks may be considered, followed by either monitoring or the implementation of long-term oral therapy. Extraction of CIEDs should be conducted in specialized medical centers [8,11].

Residual leads increase the risk of chronic infection due to biofilm formation, highlighting the importance of complete extraction, even in the absence of definitive lead involvement, particularly in cases of valvular IE, either by the transvenous route whenever possible or otherwise with or without CPB through sternotomy, thoracotomy, or thoracoscopy as mini-invasive surgical procedures, as they may lead to IE, IM, severe valvular destruction, or embolic complications, including pulmonary embolism due to septic emboli, migration of vegetations, or fragments of the lead, which can be rapidly fatal [8,12]. Indeed, open cardiothoracic and laparoscopic lead removal is recommended for chronically entrapped or embedded lead systems when transvenous access is not feasible or unsafe and often unsuccessful, with a high incidence of severe complications due to the risk of damaging vital cardiac or vascular structures. These procedures allow for safe device removal under direct visualization, often with CPB and the assistance of an experienced cardiothoracic surgeon, especially when foreign material is embedded in the endocardium or nearby vessels [13]. Video-assisted minimally invasive techniques, such as thoracoscopy, can also be used in some cases, and when necessary, intraoperative localization can be performed by TTE or TEE while considering the specific pathogens involved, the surgical risks, and the need for valve intervention [14]. The risks associated with lead extraction should be carefully evaluated in the context of the patient’s individual characteristics [8]. The surgical intervention for IE focuses on the removal of infected structures, the restoration of cardiac function, and the collection of tissue samples to inform antibiotic treatment decisions. It is recommended that the duration of antibiotic therapy be extended by an additional four to six weeks in cases presenting with septic emboli or prosthetic valve involvement [8].

This is a rare case of residual ICD lead material embedded in the right ventricle and in the left innominate vein following multiple attempts at percutaneous and surgical extractions. Patients with a residual ventricular lead from an ICD or pacemaker, after failure of complete transvenous or surgical extraction, are usually asymptomatic as long as the lead remains sterile [13]. In our case, the extractions were performed via sternotomy without CPB for the cut material trapped in the left innominate vein and under CPB with fluoroscopic guidance for the lead tip embedded in the right ventricle, as localization by TEE was not possible due to the esophageal achalasia being treated in our patient.

Several case reports of foreign bodies inside the heart have been reported in the literature. However, most were performed after opening the chest with or without CPB [13].

The Partial Oral Treatment of Endocarditis (POET) trial showed that after an initial IV treatment phase, up to 20% of patients can complete their treatment with oral antibiotics. The treatment of IE is divided into two phases: two weeks of IV treatment during which surgery and removal of infected foreign bodies are performed, followed, if the patient is stable, by six weeks of either oral or IV treatment. Rifampin is used in foreign body infections after an effective antibiotic therapy phase [8]. Daptomycin is recommended for staphylococcal and enterococcal endocarditis in combination with other antibiotics. Outpatient parenteral antibiotic therapy (OPAT) is a safe alternative, particularly for elderly patients [8].

(18F)-fluorodeoxyglucose (FDG) PET/CT and WBC single-photon emission computed tomography (SPECT)/CT are recommended for suspected prosthetic valve endocarditis (PVE) and CIED-associated IE when echocardiography is inconclusive. A meta-analysis reported 86% sensitivity and 84% specificity for (18F)-FDG PET/CT, highlighting its incremental diagnostic value alongside WBC SPECT/CT [8]. FDG-PET/CT is highly sensitive and specific for diagnosing CIED-related endocarditis, particularly in cases without pocket infection, but caution is needed for recently implanted devices (<6 weeks) [15]. WBC SPECT/CT is an alternative to PET/CT for diagnosing IE, with a sensitivity ranging from 64% to 90% and specificity from 36% to 100%, with diagnostic accuracy improved in the presence of periprosthetic abscesses [8]. However, it is limited in availability. A chest CT scan is recommended to evaluate pulmonary complications, though it carries risks for renal insufficiency. 99mTc-HMPAO-SPECT/CT has reduced diagnostic errors in IE by 27%, clarifying cases previously classified as possible IE [16]. In native valve endocarditis, PET/CT and SPECT/CT have low sensitivity but high specificity due to a lower inflammatory response compared to PVE. Whole-body (18F)-FDG PET/CT is valuable for detecting distant lesions, mycotic aneurysms, and infection entry points in patients with suspected or confirmed IE. It helps identify septic emboli in the spleen, lungs (right-sided IE), kidneys, and metastatic infections in bones, joints, muscles, and liver. However, it is less effective for detecting cerebral septic embolism and mycotic aneurysms in intracerebral arteries due to high physiological (18F)-FDG uptake in the brain [8].

Traditionally, the preoperative localization of cut leads from a cardiac device embedded in the myocardium is performed using chest radiology, including fluoroscopy, X-rays, and CT scans, as well as TTE or TEE.

The use of intraoperative TEE in lead extraction is well-documented in the literature and remains essential for suspected CIED-related IE, particularly for assessing the extent of infection, identifying complications such as thrombi or perforations, examining vegetation, and re-assessing heart valves and biventricular function before device extraction and valve repair/replacement, directly influencing surgical decisions. Post-surgery, it is mandatory to assess the immediate outcome and establish a baseline for future follow-up comparisons. However, its use is contraindicated or risky in certain cases (e.g., in the presence of severe esophageal conditions), as was the case with our patient, which leads to the consideration of other approaches such as fluoroscopy, intracardiac echocardiography (ICE), CT scans, and PET/CT to facilitate locating the lead’s exact position [8].

Intraoperative echocardiography is recommended in all cases of IE requiring surgery [8].

Fluoroscopy provides precise and iterative real-time visualization of internal structures, but it has limitations, including radiation exposure and the inability to visualize non-radiopaque objects, which can be problematic in complex extractions or infections involving soft tissues and biofilms [17]. Intraoperative imaging, such as fluoroscopy, is crucial for the precise localization of instruments, emphasizing the importance of thorough preoperative assessment and collaboration between specialists for optimal care. It plays a key role in extracting deeply embedded leads, especially in complex cases, and improves patient outcomes, particularly when TEE is contraindicated, by providing dynamic feedback to the surgeon to locate embedded leads, guiding tool placement, and ensuring complete extraction of fragments. This reduces the risk of infection and major complications such as perforations and vascular injuries while minimizing damage to surrounding tissues [18]. In our case, we believe that fluoroscopy, as an alternative tracing technique, provides valuable information for choosing the surgical approach. It allows for the identification of the lead tip position after sternotomy under CPB and facilitates its extraction after localization.

In cases of severe esophageal pathologies, such as advanced achalasia making TEE inappropriate, the combined approach of imaging using fluoroscopy, TTE, and intraoperative echocardiography and multidisciplinary surgical planning provides more effective guidance for lead extraction, reducing the risk of lead fragment migration, which can lead to severe complications [18]. TTE is less invasive than TOE, but its resolution may be insufficient in patients with complex anatomy or obesity, and it is not applicable during conventional sternotomy procedures [18].

The available data mainly come from case studies, case series, or retrospective articles, which justifies the use of the expression "in most reported cases" [19]. For example, in the case of using fluoroscopy for intracardiac lead extraction, several studies report its effectiveness in specific cases, particularly when other techniques are contraindicated [20]. However, current guidelines lack details on complex or atypical situations.

Rigorous preoperative planning and a multidisciplinary approach (endocarditis team decision) involving cardiologists, cardiothoracic surgeons, infectious disease specialists, and anesthesiologists are essential for these procedures to ensure comprehensive care. Each specialty provides a unique contribution to device management, surgical extraction, infection treatment, and anesthetic safety. Thus, the early removal of these residual devices has been recommended to prevent further damage to the heart. However, there is no consensus on the timing or surgical procedures for the removal of non-infected cut leads from a cardiac device embedded in the myocardium, particularly for patients dependent on ICD therapy, nor on the timing of reimplantation of CIEDs after their removal following infection [8].

## Conclusions

The multimodal imaging approach, incorporating echocardiography, CT, MRI, and PET/CT, enhances diagnostic accuracy for CIED-related IE. FDG-PET/CT is highly sensitive and specific, particularly when microbiological tests are inconclusive. It helps in detecting distant lesions, mycotic aneurysms, and infection entry points, as well as identifying septic emboli in organs such as the spleen, lungs, kidneys, and liver. This imaging strategy is essential for guiding treatment decisions in complex cases with unclear bacteriological results. TEE and TTE are preferred tools for lead extraction, providing real-time imaging. TEE is ideal for assessing cardiac structures and detecting complications, but it was contraindicated in this case due to severe achalasia. Fluoroscopy was therefore used as a valuable alternative, allowing real-time visualization of embedded lead tips and ensuring safe extraction. However, it has limitations, including radiation exposure and the inability to visualize non-radiopaque objects. A multidisciplinary team, along with imaging techniques such as fluoroscopy, is essential to ensure a safe and effective lead extraction. Current guidelines inadequately address the management of contraindications to TEE and complex cases involving chronic infections on residual leads requiring a more detailed approach, particularly for cases needing alternative imaging. This case emphasizes the diagnostic challenges of CIED-related infections and the importance of integrating clinical, paraclinical, and intraoperative findings, especially when bacteriological tests are inconclusive. It highlights the need for early lead extraction and a multidisciplinary approach to prevent chronic infections and complications. Future guidelines should provide strategies for managing such cases to achieve optimal outcomes.

## References

[1] Toriello F, Saviano M, Faggiano A (2022). Cardiac implantable electronic devices infection assessment, diagnosis and management: a review of the literature. J Clin Med.

[2] Qian H, Song H, Li Y, Jiang C (2015). Removal of metallic foreign body in heart by minimally invasive procedure under the guidance of transesophageal echocardiography and transthoracic echocardiogram. J Thorac Dis.

[3] Chand AR, Sarju R, Kumar SV, Singh WG (2013). Removal of a foreign body from the heart under transesophageal echocardiographic guidance. J Cardiothorac Vasc Anesth.

[4] Lakkireddy DR, Segar DS, Sood A (2023). Early lead extraction for infected implanted cardiac electronic devices: JACC review topic of the week. J Am Coll Cardiol.

[5] Li JS, Sexton DJ, Mick N (2000). Proposed modifications to the Duke criteria for the diagnosis of infective endocarditis. Clin Infect Dis.

[6] Botelho-Nevers E, Thuny F, Casalta JP (2009). Dramatic reduction in infective endocarditis-related mortality with a management-based approach. Arch Intern Med.

[7] Fowler VG Jr, Li J, Corey GR (1997). Role of echocardiography in evaluation of patients with Staphylococcus aureus bacteremia: experience in 103 patients. J Am Coll Cardiol.

[8] (2024). Correction to: 2023 ESC guidelines for the management of endocarditis: developed by the task force on the management of endocarditis of the European Society of Cardiology (ESC) endorsed by the European Association for Cardio-Thoracic Surgery (EACTS). Eur Heart J.

[9] McElhinney DB, Zhang Y, Aboulhosn JA (2021). Multicenter study of endocarditis after transcatheter pulmonary valve replacement. J Am Coll Cardiol.

[10] Snipsøyr MG, Ludvigsen M, Petersen E, Wiggers H, Honoré B (2016). A systematic review of biomarkers in the diagnosis of infective endocarditis. Int J Cardiol.

[11] Mateos Gaitán R, Boix-Palop L, Muñoz García P (2020). Infective endocarditis in patients with cardiac implantable electronic devices: a nationwide study. Europace.

[12] Mulpuru SK, Madhavan M, Asirvatham SJ, Swale MJ, Hayes DL, Friedman PA (2021). Implanting and extracting cardiac devices: technique and avoiding complications. Cardiac Pacing, Defibrillation and Resynchronization: A Clinical Approach, Fourth Edition.

[13] Tułecki Ł, Czajkowski M, Targońska S (2022). The role of cardiac surgery in transvenous lead extraction. A high-volume center experience with 3207 procedures. Kardiochir Torakochirurgia Pol.

[14] Ramadan MS, Gallo R, Patauner F, Bertolino L, Durante-Mangoni E (2022). Emerging concepts on infection of novel cardiac implantable devices. Rev Cardiovasc Med.

[15] Wang TK, Sánchez-Nadales A, Igbinomwanhia E, Cremer P, Griffin B, Xu B (2020). Diagnosis of infective endocarditis by subtype using (18)F-Fluorodeoxyglucose positron Emission tomography/computed tomography : a Contemporary meta-analysis. Circ Cardiovasc Imaging.

[16] Holcman K, Szot W, Rubiś P (2019). 99mTc-HMPAO-labeled leukocyte SPECT/CT and transthoracic echocardiography diagnostic value in infective endocarditis. Int J Cardiovasc Imaging.

[17] Berbenetz NM, Golian M, Sadek MM (2024). Preoperative and intraoperative imaging during transvenous lead extraction. Card Electrophysiol Clin.

[18] Bracke FA, Meijer A, van Gelder B (2004). Reflections of six years of lead extraction: influence on indications and technique. Neth Heart J.

[19] Bongiorni MG, Burri H, Deharo JC (2018). 2018 EHRA expert consensus statement on lead extraction: recommendations on definitions, endpoints, research trial design, and data collection requirements for clinical scientific studies and registries: endorsed by APHRS/HRS/LAHRS. Europace.

[20] Wilkoff BL, Love CJ, Byrd CL (2009). Transvenous lead extraction: Heart Rhythm Society expert consensus on facilities, training, indications, and patient management: this document was endorsed by the American Heart Association (AHA). Heart Rhythm.

